# Retraction Notice to: lncRNA lnc-TSI Inhibits Metastasis of Clear Cell Renal Cell Carcinoma by Suppressing TGF-β-Induced Epithelial-Mesenchymal Transition

**DOI:** 10.1016/j.omtn.2022.08.010

**Published:** 2022-08-16

**Authors:** Peng Wang, Weixiong Chen, Tongtong Ma, Zhaoyu Lin, Chongbin Liu, Youhua Liu, Fan Fan Hou

(Molecular Therapy: Nucleic Acids *22*, 1–16; December 2020)

This article has been retracted at the request of the first author and editor-in-chief.

The first author contacted the journal to report image misrepresentation and requested to withdraw the article. The author reported duplicated images in Figures 5A, 5B, and S3A. Image analysis performed by the editorial office confirmed these image duplications and identified additional duplicated images in Figure 6A. This reuse (and in part misrepresentation) of data without appropriate attribution represents a severe abuse of the scientific publishing system. The authors apologize for any inconvenience it may cause.

